# Outcomes of Functional Endoscopic Sinus Surgery in Chronic Rhinosinusitis: A Systematic Review and Meta-Analysis

**DOI:** 10.7759/cureus.53952

**Published:** 2024-02-10

**Authors:** Saad Algahtani, Abdullah Alhajlah, Abdullah I Abuharb, Abdullah F Alzarroug, Alwaleed I Almughira, Nasser Alsywina, Faris K Alahmadi, Sami Al-Dubai

**Affiliations:** 1 College of Medicine, Imam Mohammad Ibn Saud Islamic University, Riyadh, SAU; 2 College of Medicine, King Saud Bin Abdulaziz University for Health Sciences, Riyadh, SAU; 3 Joint Program of Preventive Medicine Post Graduate Studies, Ministry of Health, Medina, SAU

**Keywords:** fess, quality of life, middle east, functional endoscopic sinus surgery, chronic rhinosinusitis

## Abstract

Chronic rhinosinusitis (CRS) is a prevalent health problem that affects many people around the world and can require surgical intervention if conservative therapy fails. Functional endoscopic sinus surgery (FESS) is a minimally invasive surgical procedure commonly used to manage CRS. The success of FESS depends on various factors, and larger studies are necessary to determine its efficacy in managing CRS in this population. This systematic review and meta-analysis of the available literature aims to provide a comprehensive assessment of the effectiveness of FESS in the Middle East. We followed the standards outlined by PRISMA and the Cochrane Handbook for systematic reviews. The primary outcome of interest was the quality of life (QOL), and the secondary outcome was the recurrence of CRS. This systematic review and meta-analysis was conducted, and sensitivity analysis was performed to examine the robustness of the results. Six studies were included. The review found that the QOL significantly improved (p < 0.001). Two studies reported recurrence of CRS after FESS, and data showed that the recurrence of CRS after surgery was 6%. FESS is an effective intervention for CRS, but further research is needed on recurrence rates.

## Introduction and background

Chronic rhinosinusitis (CRS) is a common health problem that affects millions of people around the world, with a prevalence of up to 10% in some populations [1]. CRS is characterized by inflammation of the paranasal sinuses and nasal mucosa and is associated with symptoms such as nasal congestion, facial pain, and headache [2]. Despite the availability of various medical treatments, some patients with CRS may not respond to conservative therapy and may require surgical intervention. Functional endoscopic sinus surgery (FESS) is a minimally invasive surgical procedure commonly used to manage CRS [3]. The procedure aims to restore normal sinus ventilation and drainage by removing inflamed tissue and obstructions within the sinuses, thus improving symptoms and quality of life [4]. Although FESS effectively manages CRS, the outcomes of FESS in the Middle East and North Africa (MENA) remain largely undetermined. The Saudi population, for example, has unique demographic and cultural characteristics, and the prevalence of CRS in this population has been reported to be as high as 13.9% [5].

Various factors influence the management of CRS in the Middle East. One of the critical factors is the lack of awareness among the general public about the disease and its management. Studies have shown that a large population in the Middle East lacks knowledge about CRS, which can lead to delays in seeking medical attention and proper treatment [6]. The availability and accessibility of healthcare services have also affected the manner of CRS management in the region. As a result, patients in the region often have to endure delayed diagnosis and treatment, which can further worsen the condition of patients with CRS.

Despite the challenges, FESS has delivered reliable results as an effective treatment option for patients with CRS. A primary outcome observed in most experimental trials was the high rate of improvement in symptoms and quality of life (QOL). A study conducted in 2006 on 47 patients who underwent FESS for CRS found that 87.2% of the patients reported a significant improvement in their symptoms after surgery and 72.4% reported an improvement in their QOL [7]. Similarly, Bunzen et al. experimented on 58 patients. They reported similar findings, with 86.2% of patients reporting an improvement in their symptoms and 77.6% reporting an improvement in their QOL [8]. Alrajhi et al. also reported a significant improvement in CRS symptoms, as observed in 60 CRS patients undergoing FESS. Symptoms such as nasal obstruction, post-nasal drip, and facial pain were reported to improve significantly. The patient's QOL was also reported to show a marked improvement post-surgery [9]. These positive outcomes of FESS are common among Middle Eastern CRS patients. Al-Qudah et al. [10] investigated another group of 78 patients and reported that FESS significantly improved the symptoms of CRS. Through self-reported data, the researchers found that patients significantly improved their QOL post-surgery. The study also reported a low complication rate and no significant adverse events [10].

However, it is important to note that the success of FESS depends on various factors, including the severity of the disease, the extent of surgery, and patient factors such as smoking status and adherence to postoperative care. Studies in the Middle East have also reported positive outcomes of FESS in patients with CRS. However, a common limitation in most of these studies remains the lack of appropriate patient selection and unreliable preoperative management. A larger pool of patients is necessary to assess the outcomes of FESS in the Middle Eastern population to determine the efficacy of this procedure in the management of CRS in this demographic. The primary purpose of this review is to evaluate the available evidence on the outcomes of FESS in the treatment of CRS in the Middle East, including its efficacy, safety, and patient satisfaction. The findings of this review can help inform clinical practice and improve the quality of care for CRS patients in the Middle East.

## Review

Methods

Study Design

This systematic review and meta-analysis was conducted in accordance with the standards of systematic reviews outlined by the Preferred Reporting Items for Systemic Reviews and Meta-Analyses (PRISMA). It used the PRISMA extension published in the Cochrane Handbook for systematic reviews and interventions [11].

Literature Search

An extensive literature search was performed from relevant electronic databases, such as PubMed, Cochrane Library, and Scopus. A combination of Medical Subject Headings (MeSH) and keywords related to FESS and CRS were used. Examples of primary keywords used to construct the basic search string were "functional endoscopic sinus surgery" OR "FESS", "chronic rhinosinusitis" OR "CRS," "Middle East” OR “MENA”. The names of the Middle Eastern countries were also included in the search.

Inclusion and Exclusion Criteria

Two independent investigators participating in the systematic review and meta-analysis reviewed the identified studies. The inclusion criteria focused on studies that evaluated the outcomes of FESS in the treatment of CRS and studies that reported quantitative data on outcomes such as symptom improvement, QOL, or recurrence rates. All selected studies should be experimental studies that were conducted in the MENA region and published in English. Middle Eastern studies published in other languages, but were translatable into English, were included.

Studies concerning patients with acute sinusitis or other sino-nasal disorders were excluded. Similarly, other surgical techniques, besides FESS, were grounds for exclusion. Priority was given to RCTs for inclusion, as well as cohort studies (CH) and method cohort (MCH), and other study designs were excluded.

Quality Assessment

The quality of the studies included in this meta-analysis was assessed using the Newcastle-Ottawa scale (NOS) for non-randomized studies. The NOS assesses the quality of non-randomized studies based on a selection of participants, comparability of groups, and outcome assessment. Studies will be considered of high quality if they have a low risk of bias or a high score on the NOS.

Data Extraction

Before extracting the data, the included studies were evaluated for risk of bias according to the methodological standards outlined in the Cochrane Handbook of Systematic Reviews of Interventions [11]. A standardized Excel sheet was prepared and refined with the purpose of extracting data that would be relevant for this systematic review and meta-analysis. The investigators involved in selecting the studies also extracted relevant information for this review. Data extracted included study identification (such as author and year of publication), study characteristics (such as study design, sample size, and follow-up period), patient characteristics (such as age, sex, and comorbidities), details of the FESS intervention (such as technique and intraoperative complications), and outcome measures (such as efficacy, safety, and patient satisfaction).

Data Analysis

A meta-analysis was performed using a random-effects model to pool the results of eligible studies. Heterogeneity was assessed using the I2 statistic. A level of heterogeneity ≤ 25% was tolerable to ensure that the included studies were homogeneous. The significance of the test shall be determined by the p-value, with the point of statistical significance being ≤ 0.05. Sensitivity analyses were performed using a fixed-effects model analysis. Data were analyzed and plotted on Review Manager software (RevMan 5.4; Cochrane Collaboration, London, UK).

Results 

Study Selection

**Figure 1:**
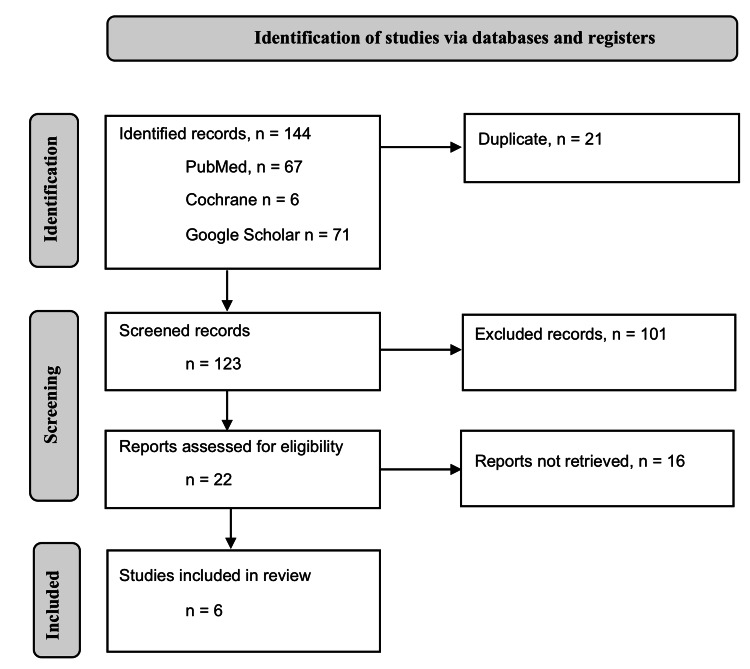
PRISMA flow diagram to illustrate the study selection process.

The initial literature search in the databases revealed 144 qualitative studies for inclusion in the systematic review and meta-analysis. Twenty-one studies from these studies were duplicates, which were eliminated, leaving 123 studies for further eligibility assessment. The title and abstract assessment of the remaining studies eliminated 101 articles. A full-text assessment of the 22 remaining studies resulted in the elimination of 16 articles. The remaining six studies were extracted for data (Table 1). The PRISMA flow diagram below (Figure 1) was then developed to illustrate the study selection process.

**Table 1:** Study characteristics of the selected studies.

Author	Year	Study Design	Location	Sample Size	Age	Sex	Comorbidities	Follow-up Period	Quality of Life (Mean ± SD)	Recurring CRS	Duration Before Symptoms Recurrence	Recurring Symptoms
Alghonaim et al. [12]	2020	Retrospective Cohort Study	Saudi Arabia	28	13-55 (Mean: 31.57 years)	15 (53%)F + 13 (46%)M	18 patients (18.2%) had asthma and 13 patients (13.1%) had allergic rhinitis.	6 to 12 months. Mean follow-up of 50.5 months.	N/A	8	1 year	Patients with disease recurrence had higher occurrence rates of symptoms such as nasal discharge (87.5%), post-nasal drip (37.5%), facial pressure/pain (50%), headache (50%), nasal polyposis (87.5%), hypertrophy of inferior turbinate (37.5%), and proptosis (12.5%).
Al Sharhan et al. [13]	2021	Longitudinal Prospective Study	Saudi Arabia	68	19-50 (Mean age 36.58 years)	37 (54.41%)M + 31 (45.59%)F	N/A	12 months	18.12 ± 8.42	35	19 weeks	Recurrent symptoms were nasal obstruction (87.1%), nasal discharge (80%), and facial pain/pressure (80%). As well as decreased sense of smell (54.3%), post-nasal drip (54.3%), and cough (37.1%).
Qadeer et al. [14]	2018	Prospective Study	Saudi Arabia	54	Mean (SD): 35.98 (7.68) Years	32M + 22F	Asthma was present in 12 patients	13 months	39.50 ± 20.75	4	N/A	N/A
Behiry et al. [15]	2019	Prospective Study	Egypt	60	25-40 (Mean 32.9±5.3 years)	34M + 26F	N/A	3 months	2.536 ± 0.98	Outcome: FESS significantly improved disease-specific quality of life as measured by the Sino-Nasal Outcome Test-22 questionnaire score with a p-value of less than or equal to 0.001.		
Aghdas et al. [16]	2018	Prospective Study	Iran	59	40.88 ± 16.11 years	38M + 21F	N/A	N/A	35.37 ± 11.41	Outcome: The statistical analysis revealed that endoscopic surgery led to a significant reduction in SNOT-22 questionnaire scores (P < 0.000). A positive correlation between the pre-operative score and the post-operative gain (Spearman correlation coefficient: 0.419, P: 0.001). Higher pre-operative scores resulted in greater improvement after surgery.		
Laababsi et al. [17]	2019	Prospective Cohort Study	Morocco	66	38.96 ± 13.58 years	31M + 35F	Asthma and smoking	6 months	23.05 ± 9.01	Outcome: The study found that FESS improves all domains of QOL in patients.		

Quality Assessment

For the six non-RCTs included in this investigation, the NOS appraisal system was used to perform a quality assessment of the involved studies (Table 2) [18]. The scores for each item are then summed to give an overall score for the study. The maximum score on the NOS is 9, with higher scores indicating better study quality.

**Table 2:** Results of the quality assessment of the six non-randomized studies according to the NOS.

Author (year)	Selection				Comparability		Outcome			Score
	Representative of Exposed Cohort	Selection of Non-exposed Cohort	Ascertainment of Exposure	Demonstration That Outcome of Interest Was not Present at the Start of Study	Control for Important Factors	Additional Factors	Assessment of Outcome	Follow-Up	Adequacy of Follow-Up	
Alghonaim et al. [12]	Yes	Yes	Yes	Yes	No	No	Yes	Yes	Yes	8
Al Sharhan et al. [13]	Yes	Yes	Yes	Yes	Yes	Yes	Yes	Yes	Yes	9
Qadeer et al. [14]	Yes	Yes	Yes	Yes	No	Yes	Yes	Yes	Yes	8
Behiry et al. [15]	Yes	Yes	Yes	Yes	No	No	Yes	Yes	No	6
Aghdas et al. [16]	Yes	Yes	Yes	Yes	Yes	No	Yes	No	No	6
Laababsi et al. [17]	Yes	Yes	Yes	Yes	Yes	No	Yes	Yes	Yes	8

Statistical Analysis

**Figure 2:**
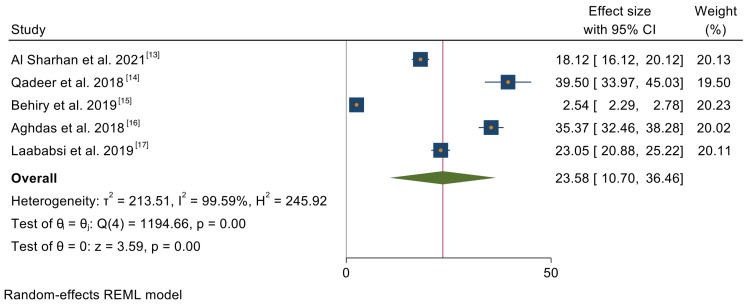
A forest plot of the meta-analysis on the QOL of CRS patients after FESS.

QOL: The QOL outcome was evaluated by five studies. The mean difference in QOL was 23.58 (10.70, 36.46) at a 95% confidence interval. The overall effect of z = 3.59 (p < 0.001) was statistically significant in the change in QOL outcome among patients with CRS undergoing FESS. The level of heterogeneity was high (I2 = 99.59%, p < 0.001). The results of this meta-analysis are reported in Figure 2.

**Figure 3:**
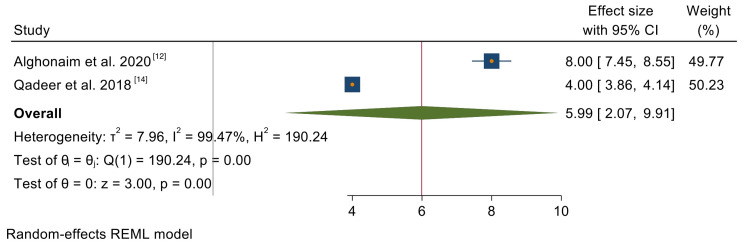
A forest plot of the meta-analysis on the recurrence of CRS after FESS.

Recurring CRS: Only two of the six included studies reported on the recurrence of CRS after FESS. The analyzed studies were found to have high heterogeneity (I2 = 99.47%, p = 0.00). There was an overall recurrence rate of 6% (95% CI = 2.07, 9.91). The results of this analysis are represented in Figure 3.

Discussion

Six studies were included in this systematic review and meta-analysis, with 335 patients observed. All 335 patients were diagnosed with some form of sino-nasal disease, CRS being the common point of interest. Our aim was to determine the outcome of FESS as an intervention for CRS. The review observed the change in QOL as tested by the Sino-nasal outcome test (SNOT-22) questionnaire and the CRS recurrence rate after surgery.

Only three studies reported the recurrence of CRS [12-14]. The data collected indicate an overall CRS recurrence rate of 47/150 (31.33%) after an average follow-up period of nine months. With a p-value of p = 0.11, there was no statistical significance in the recurrence of CRS after FESS. Before Alghonaim et al., multiple studies in the Middle East found that CRS symptoms recur at a high rate after FESS [12]. Telmesani [19] and Marglani et al. [20] reported 54.4% recurrence in the eastern province of Saudi Arabia and 55% in Makkah. Following this study, Al Sharhan et al. reported many recurring CRS symptoms in 35/68 (51.47%) patients [13]. A year after FESS, in patients diagnosed with CRS with nasal polyps (CRSwNP), the presence of nasal polyps contributed to recurrence. Smith et al. estimated the occurrence of such an event to be 20%. The study also notes a 2.1 likelihood of improvement for patients undergoing surgery [21].

Five studies included in this systematic review and meta-analysis used SNOT-22 primarily to assess QOL. Al Sharhan et al. went into depth to explain the underlying elements that led to FESS being a physician's choice of intervention [13]. Other investigations take a more direct approach. For instance, Qadeer et al. demonstrated a gradual improvement in the mean score of QOL over a one-year follow-up period. The QOL score is reduced from 52.31 to 13.69, 11.26, 12.5, and 12.81 in postoperative 1st, 3rd, 6th, and 12th months, respectively [14]. A similar trajectory has been replicated in other studies outside the Middle East, such as Jayakrishna et al., who reported a mean change of 33.04 with p < 0.001, indicating a high significance [22]. Preoperative factors such as smoking, allergies, and asthma were predictors of a poor QOL score after FESS. A study conducted in India opined that smoking, alcoholism, and snuff use predict the outcome of symptom recurrence and QOL [23].

Primarily, with FESS, the disease-specific QOL can be significantly improved. Three separate studies demonstrate this finding in Egypt by Behiry et al. [15], Iran by Aghdas et al. [16], and Morocco by Laababsi et al. [17]. A significant improvement has been observed in the QOL scores between preoperative and postoperative observations [17]. Similarly, a mean score of QOL of 59.38 ± 5.84 was observed before the operation and improved to 24.01 ± 10.48 after surgery. This reduction has been significant across the board, regardless of location. Statistical results indicate a level of significant improvement of p < 0.001. 

Symptom pattern improvement and QOL are two highly interrelated outcomes in surgery patients. After four quarterly post-surgery observations, Sujatha et al. found positive treatment responses to FESS with a mean change of 75 SNOT score [23]. FESS has a positive impact on sleep outcomes. Therefore, patients with CRS often complain of excessive sleepiness and fatigue during the day. Since CRS affects the local area and the body as a whole, and FESS reduces the burden of the disease by improving systemic cytokine levels, it is reasonable to assume that FESS would also lead to improved sleep outcomes [15]. These symptom improvements inform the overall QOL of the patients under treatment. As a result, more than 80% of patients with co-existing sinusitis and asthma also showed a moderate-to-great improvement in symptoms [23]. Improving QOL can be reinforced by increased postoperative care, such as topical steroids. Although QOL can improve with or without irrigation with budesonide, there is a statistically significant improvement in efficacy and safety.

## Conclusions

This systematic review and meta-analysis of six studies aimed to determine the outcome of FESS as an effective intervention for the management of CRS in the Middle Eastern population. The review found that a significant majority of patients undergoing FESS reported substantial improvement in their symptoms and an improved overall QOL. This is evident from the reduction in SNOT-22 scores postoperatively, indicating better health outcomes. However, it is crucial to note the variability in recurrence rates of CRS post-FESS, which highlights the need for careful patient selection and management, including considerations of preoperative comorbidities. Nevertheless, there is a need for larger-scale studies and standardized preoperative and postoperative management protocols to further validate these findings and optimize patient outcomes.
